# Supplementary material for: Deletion of wfs1 Impairs Oligodendrocyte Precursor Cells Dorsal Distribution and Myelination Through the wfs1-hmgcs1 Axis in Zebrafish
Source: Biology (Basel). 2026 Aug 16;15(16):1402. doi: 10.3390/biology15161402 (PMC13510151; doi:10.3390/biology15161402)
Supplement: Supplementary file 1 [file biology-15-01402-s001.zip › File S1-Transcriptome sequencing data and differentially expressed genes (DEGs) in wfs1-- zebrafish at 5 dpf/1.GO/wfs1KOvsWT/DOWN/wfs1KOvsWT.down.GObp_DAG.pdf]

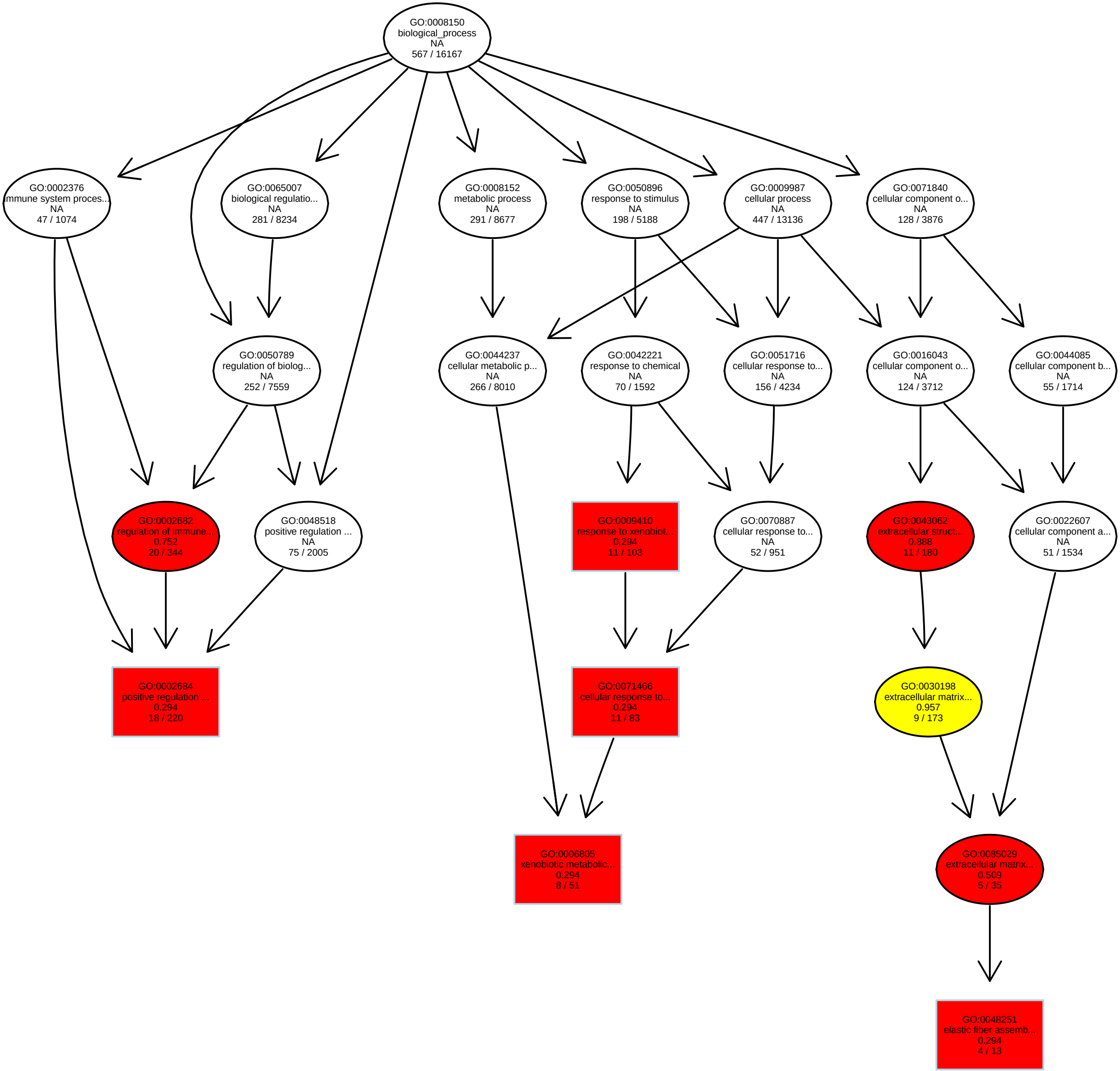

GO:0008150
biological_process
NA
567 / 16167
GO:0002376
immune system proces...
NA
47 / 1074
GO:0065007
biological regulatio...
NA
281 / 8234
GO:0008152
metabolic process
NA
291 / 8677
GO:0050896
response to stimulus
NA
198 / 5188
GO:0009987
cellular process
NA
447 / 13136
GO:0071840
cellular component o...
NA
128 / 3876
GO:0050789
regulation of biolog...
NA
252 / 7559
GO:0044237
cellular metabolic p...
NA
266 / 8010
GO:0042221
response to chemical
NA
70 / 1592
GO:0051716
cellular response to...
NA
156 / 4234
GO:0016043
cellular component o...
NA
124 / 3712
GO:0044085
cellular component b...
NA
55 / 1714
GO:0002682
regulation of immune...
0.752
20 / 344
GO:0048518
positive regulation ...
NA
75 / 2005
GO:0009410
response to xenobiot...
0.294
11 / 103
GO:0070887
cellular response to...
NA
52 / 951
GO:0043062
extracellular struct...
0.888
11 / 180
GO:0022607
cellular component a...
NA
51 / 1534
GO:0002684
positive regulation ...
0.294
18 / 220
GO:0071466
cellular response to...
0.294
11 / 83
GO:0030198
extracellular matrix...
0.957
9 / 173
GO:0006805
xenobiotic metabolic...
0.294
8 / 51
GO:0085029
extracellular matrix...
0.509
5 / 35
GO:0048251
elastic fiber assemb...
0.294
4 / 13
